# Cd-Resistant Strains of *B*. *cereus* S5 with Endurance Capacity and Their Capacities for Cadmium Removal from Cadmium-Polluted Water

**DOI:** 10.1371/journal.pone.0151479

**Published:** 2016-04-14

**Authors:** Huiqing Wu, Qingping Wu, Guojie Wu, Qihui Gu, Linting Wei

**Affiliations:** 1 State Key Laboratory of Applied Microbiology Southern China, Guangdong Institute of Microbiology, Guangzhou, Guangdong, China; 2 Guangdong Provincial Key Laboratory of Microbial Culture Collection and Application, Guangdong Open Laboratory of Applied Microbiology, Guangzhou, Guangdong, China; 3 College of Chemistry and Chemical Engineering, Zhongkai University of Agriculture and Engineering, Guangzhou, Guangdong, China; 4 Guangdong Huankai Microbial Sci. & Tech. Co., Ltd., Guangzhou, 510663, China; Friedrich Schiller University, GERMANY

## Abstract

The goal of this study was to identify Cd-resistant bacterial strains with endurance capacity and to evaluate their ability to remove cadmium ions from cadmium-polluted water. The *Bacillus cereusS5* strain identified in this study had the closest genetic relationship with *B*. *cereus* sp. *Cp1* and performed well in the removal of Cd^2+^ions from solution. The results showed that both the live and dead biomasses of the Cd^2+^-tolerant *B*. *cereus* S5 strain could absorb Cd^2+^ ions in solution but that the live biomass of the *B*. *cereus S5* strain outperformed the dead biomass at lower Cd^2+^concentrations. An analysis of the cadmium tolerance genes of *B*. *cereus S5* identified ATPase genes that were associated with cadmium tolerance and involved in the ATP pumping mechanism. The FTIR spectra revealed the presence of amino, carboxyl and hydroxyl groups on the pristine biomass and indicated that the cadmium ion removal ability was related to the structure of the strain. The maximum absorption capacity of the *B*. *cereus* S5 strain in viable spore biomass was 70.16 mg/g (dry weight) based on a pseudo-second-order kinetic model fit to the experimental data. The Langmuir and Langmuir-Freundlich isotherm adsorption models fit the cadmium ion adsorption data well, and the kinetic curves indicated that the adsorption rate was second-order. For Cd^2+^ concentrations (mg/L) of 1–109 mg/L, good removal efficiency (>80%) was achieved using approximately 3.48–10.3 g/L of active spore biomass of the *B*. *cereus* S5 strain. A cadmium-tolerant bacteria-activated carbon-immobilized column could be used for a longer duration and exhibited greater treatment efficacy than the control column in the treatment of cadmium-polluted water. In addition, a toxicity assessment using mice demonstrated that the biomass of the *B*. *cereus* S5 strain and its fermentation products were non-toxic. Thus, the isolated *B*. *cereus S5* strain can be considered an alternative biological adsorbent for use in emergency responses to severe cadmium pollution and in the routine treatment of trace cadmium pollution.

## Introduction

With the rapid development of the economy, environmental pollution, including the discharge of heavy metal-contaminated wastewater, is becoming increasingly serious. Many water bodies, such as rivers, lakes and reservoirs, which often serve as sources of drinking water, have been gradually polluted by heavy metals, especially in developing countries [1–2]. In addition, sudden accidents have also caused serious water pollution. This pollution leads to serious ecological and health hazards because of its severe toxic effects. Among heavy metals, cadmium is one of the most toxic. Cadmium is a group B1 carcinogen, targeting the lungs, liver, and kidneys, and prolonged exposure can damage renal function and cause tumour growth. Its nephrotoxicity, immunotoxicity and osteotoxicity have also been documented [3]. Trace cadmium pollution of water sources is common worldwide [4–5], and, unfortunately, even relatively low doses of cadmium are extremely toxic [6–7]. Thus, there is an urgent need to develop more effective methods for removing cadmium pollutants from the environment, including drinking water sources.

To date, the treatment techniques for cadmium-polluted water mainly include physical, chemical and biological methods. Literature reports indicate that many non-living materials are also very effective as physical adsorbents for cadmium, such as industrial and agricultural by-products and other materials [8–16]. However, the large-scale application of cadmium-polluted water treatment techniques has yet to be realized because the use of non-living materials also produces large quantities of sludge, the treatment of which is difficult. These techniques are also limited by high costs, intensive labour requirements and adverse environmental effects. Chemical precipitation and electrochemical treatment, ion exchange strategies, membrane technologies, and activated carbon adsorption processes cannot be used on the large-scale when the metal ion concentration in aqueous solution is between 1 mg/L and 100 mg/L because they are ineffective or extremely expensive. Volesky (2001) summarized the advantages and disadvantages of these conventional metal removal technologies [17]. For these reasons, more cost-effective and environmentally friendly technologies must be developed for metal remediation.

As of late, biological techniques employing microbes and nutrients as heavy metal removal tools have shown the most promise [18]. Biosorption by the passive binding of heavy metals to microorganisms (e.g., bacteria, fungi, and algae) has significant potential for industrial effluent treatment due to its environmental friendliness, economic viability, good metal binding capacity, limited sludge generation, and ability to efficiently remove metals from dilute effluents [19–21]. Microbial methods, especially those using active heavy metal-tolerant microbes screened from pollution sites, might be ideal candidates for the treatment of high-volume, low-concentration complex wastewaters [22]. Microorganisms, including live and dead cells, can be used for heavy metal wastewater treatment [23]. Several types of microorganisms, including common microbial biomass and cadmium-tolerant microorganisms or endophytic microorganisms in plants, have been used in the treatment of cadmium-polluted water [24–26].

Previous studies have identified the following microorganisms as being highly cadmium-tolerant: *Bacillus laterosporus* and *Bacillus licheniformis* [27], a strain of *Paecilomyces lilacinus* [28], *Bacillus cereus RC-1* [29], *Bacillus cereus*, *Bacillus sphaericus*, *Bacillus subtilis* [30] and *Pseudomonas putida* [31]. Among these microorganisms, *Bacillus* sp., *B*. *cereus*, *B*. *sphaericus* and *B*. *subtilis* were found to have maximum cadmium bioaccumulations of 10.8 mol/g biomass, 8.0 mol/g biomass, 11.8 mol/g biomass and 9.5 mol/g biomass, respectively [30].

In summary, the active or inactivated biomass of highly cadmium-tolerant microorganisms screened from soil contamination points or cadmium-tolerant plants can effectively remove cadmium ions from solution. The examples reviewed above indicate that bioremediation using growing microbes is a feasible alternative to the purely adsorptive removal of metal contaminants from complex industrial effluents. There is a lack of reports on the use of highly cadmium-tolerant microbial agents for cadmium pollution water treatment, including emergency responses to severe cadmium pollution or the routine treatment of trace cadmium pollution. This is likely due to the special characteristics of raw water supplies, which contain a variety of trace organic and inorganic pollutants and lack nutrients. In addition, the safety of microorganism screening from a contamination standpoint has not been carefully studied.

In this study, to address the cadmium pollution of raw water and the need for a means of removing heavy metal ions in water treatment processes, cadmium-tolerant strains were screened from cadmium-polluted soil. The growth characteristics and the special adsorption properties of the cadmium-tolerant strains in different media and in water were studied. Ultimately, an effective and safe biological treatment for the removal of cadmium ions from source water was developed for application in cadmium pollution emergencies or normal trace pollution remediation.

## Materials and Methods

### Isolation of cadmium-tolerant strains from polluted soils

Polluted soil samples were collected from an area of approximately 50–100 m^2^ at the Changsha XianHe Chemical Plant in Liuyang City. The chemical plant is located on a hill near Liuyang City, which covers an area of 73.7 km^2^ and represents a total investment of 1000 million yuan. In June 2009, a serious cadmium pollution incident occurred, causing the urinary cadmium concentrations of more than 100 people as well as the Cd^2+^ ion concentrations in the soil and crops over an area of approximately 4 km^2^ to exceed the relevant standards. Septic operation procedures were used for soil collection, which was conducted at random locations around the chemical plant. This sampling did not require permission and did not involve endangered or protected species in the open space of the cadmium pollution site.

Various cadmium-tolerant microorganisms were isolated from the soil and identified at the Guangdong Institute of Microbiology. The cadmium-tolerant strains were isolated by adding approximately 1 g of cadmium-contaminated soil samples to an Erlenmeyer flask containing glass beads and 99 mL of sterilized saline water. The samples were then oscillated for 30 min at 28°C. After a 10-fold dilution, the strains were cultured in nutrient agar (NA) and potato dextrose agar (PDA) media with different concentrations of cadmium chloride (CdCl_2_·2.5H_2_O, AR, Aladdin, Shanghai, China. CAS: 7790-78-5) for 1–5 days at 37 and 30°C, respectively. The cadmium-tolerant strains were then collected.

The tolerance of the isolates to different cadmium salt concentrations was tested using the agar block method on 15–20 cm plates. Using different concentrations of cadmium salt media, the bacterial isolates were cultured in a 37°C incubator for 1–3 days, and the fungal isolates were cultured in a 30°C incubator for 2–5 days. The tolerance of the strains to cadmium chloride salt was tested by observing the growth of the microbial strains on the different concentrations of cadmium salt media.

### Cadmium ion absorption testing of the isolates

After sterilizing and cooling the 150 mL nutrient broth (NB) medium or Sabouraud's liquid medium (SB) containing different Cd^2+^ ion concentrations in 300-mL Erlenmeyer flasks, approximately 3 mL of a liquid strain was added to each liquid culture. The resistant bacteria were incubated at 33°C and stirred at 200 rpm for 39 h, and the resistant moulds were incubated at 33°C and stirred at 200 rpm for 64.5 h. Different concentrations of cadmium salt were added to the water or liquid medium to test the removal performance of the selected strains. Cadmium salt was also added to flasks that were not inoculated to serve as a control.

After centrifugation of the fermented liquid at 12,600 rpm for 2 min and dilution to the appropriate range of detection, the Cd^2+^ concentrations in the culture supernatants were measured via inductively coupled plasma mass spectrometry (ICP-MS, Agilent 1260-7700e; Agilent Technologies Co. Ltd, USA). The biosorptive capacity and the Cd^2+^ removal ratio were calculated from the following equations, respectively:

qe=(C0−Ce)X (1)

Removalratio(%)=(C0−Ce)C0×100 (2)

where q_e_ is the adsorption capacity of the biosorbent (mg/g dry cell), X is the biomass concentration (g dry cell/L), and C_0_ and C_e_ are the initial and equilibrium Cd^2+^ concentrations (mg/L), respectively.

### Cadmium removal efficiencies testing of the tolerant strains suspensions

After the viable biomass was collected via centrifugation from the resistant bacteria cultured at 37°C for 4 days (S5 and S27) and the tolerance fungi cultured at 30°C for 5 days (S47, S50 and S52), suspensions of the tolerant strains were prepared by mixing the biomass with a small amount of sterile water, and an aqueous solution of metals of known concentration (prepared from CdCl_2_ salt, respectively) was added to the biomass suspension, which was dispersed using a hand homogenizer in the tap water, using contact times of 60 min. The residual concentration of Cd^2+^ in solution was analysed as above. The absorptive capacity and the Cd^2+^ removal ratio were calculated as above.

### Growth curve and morphological observation of the cadmium-tolerant strain S5

The S5 strain was cultured in BD Bacto^TM^ tryptic soy broth (TSB; Becton, Dickinson and Co.), HKM TSB, and HKM NB (Microbial Technology Co., Ltd., China) broth, with 1%, 5% and 10% liquid cultures of the cadmium-tolerant S5 strain (150 mL TSB medium in a 500-mL conical bottle, shaken at 150 rpm, at 37°C, for 17 h), respectively. An automatic analyser (BioscreenC) with 100 pore plates was used to analyse the growth curves. The morphology of the S5 strain cultured under different conditions was observed using scanning electron microscopy.

### Identification of the cadmium-tolerant strain and the cadmium tolerance genes

The cadmium-tolerant strains were identified via the molecular identification method described in the literature [32]. After cultivation for 24 h at 37°C on tryptic soy agar (TSA), a single colony of pure isolate was inoculated into 10 mL of TSB at 37°C for 24 h (the NB, TSB, TSA, PDA and SB media used in this study were powder culture media purchased from Guangdong Province Huankai Microbial Technology Co., Ltd., Guangzhou, China). Genomic DNA was extracted using a genomic DNA extraction kit (Dongsheng Biotech, Guangzhou, China) according to the manufacturer’s instructions. The concentration of genomic DNA was determined at 260 nm using a NanoDrop-ND-1000 UV-vis spectrophotometer (Thermo Fisher Scientific, MA, USA). All oligonucleotide primers used in this study were synthesized by Beijing LiuheHuada Gene Company Ltd. The 16S rDNA PCR method was used for the bacteria, and the primers were the 27F and R1492 components (F27: 5‘–AGAGTTTGATCCGGCTCAG–3’; R1492: 5‘–TACGGCTACCTTGTTACGACTT–3’). The PCR system included a total volume of 25 μL (2 μL each of the primers F27 and R 1492 or ITS1 and ITS4, 9 μL of DDH_2_O, 2 μL of DNA and 10 μL of 2× PCR Master 'Mix) and a PCR tube.

The amplification reactions were performed in a Bio-Rad United PCR apparatus. The PCR amplification procedure involved the following reaction conditions: pre-degeneration at 95°C for 5 min; denaturation at 95°C for 30 s; annealing at 56°C for 45 s for 35 cycles; extension at 72°C for 10 min; and finally, preservation at 4°C. The amplification products were analysed by gel electrophoresis using 1.5% agarose in 1x TAE buffer with goldview dye in the electrophoresis apparatus EPS 300 (Shanghai, Tanon; Shanghai, China). Detection was permormed using the BioDocAnalyze video documentation system (GE Image Quant 350, GE Healthcare, American). After automation, DNA sequencing was performed by Beijing LiuheHuada Gene Company Ltd.

A series of similar sequences was identified by consulting the National Centre for Biotechnology Information (NCBI) database. An identification system was developed based on the construction of phylogenetic trees using the microbial organisms’ 16S rDNA nucleotide sequences by MEG5.0.

After the cadmium-tolerant strain was identified, in order to understand the tolerance mechanism of cadmium ions of the isolate, the cadmium tolerance genes were identified via molecular methods.

Because the CadA cationic drain system had previously been found to be associated with ATPase [33], the ATPase genes were investigated in the Cd-resistant strain *B*. *cereus* S5 for an association with cadmium tolerance using nested PCR technology.

The methods including the culturing and extracted genomic DNA of the cadmium-tolerant strain *B*.*cereus* S5 relied on a DNA concentration detector, the PCR apparatus, electrophoresis apparatus and the BioDocAnalyze video documentation system, the key reagents including 2× PCR Master Mix and the DL 2000 DNA marker from TakaRa (Japan). After automation, DNA sequencing was performed by Beijing LiuheHuada Gene Company Ltd.

The following three primers of the *cad*A genetic resistance to cadmium were used: CadA1, AGGATGCAAAAGTAAATTYTGGHGC; CadA2, CGYTTTGAATTTGACATGAAAACMC; and CadA3, GTGCTTCCTCTACAAGATGAATAAT.

The genomic DNA of the cadmium-tolerant strain *B*. *cereus* S5 was used as a template and CadA1 and CadA3 were used as primers for the first round of PCR amplification in separate reactions. For the second round of PCR amplification, 0.5 μL of the PCR amplification products from the first round were used as a template, and CadA2 and CadA3 were used as primers.

The 2× PCR reactions were performed in a total volume of 20 μL (1 μL of each primer, 7–7.5 μL of DDH_2_O, 0.5–1 μL of DNA and 10 μL of 2× PCR Master Mix) in a PCR tube. The PCR amplification procedure involved the following reaction conditions: pre-degeneration at 94°C for 5 min; denaturation at 94°C for 1 min, annealing at 52°C for 1 min, and extension at 72°C for 2 min for 30 cycles; and then extension at 72°C for 10 min°C.

### Preliminary toxicity testing of the cadmium-tolerant strain of *B*. *cereus* sp. *S5*

#### The animal toxicity test involving the *B*. *cereus* S5 strain

The test subjects were 15 male Chinese Kun Ming (KM) specific-pathogen-free (SPF) mice that were 20–25 days old and weighed 18–22 g (Guangdong Province Medical Animal Experimental Centre, SCXK 2013–0002). All of the animal procedures complied with “The Guide for the Care and Use of Laboratory Animals” and were approved by the Animal Care Committee of the Centre for Disease Control and Prevention of Guangdong Province (Approval ID: 13827183491). The mice were randomly divided into 3 groups (CK, A, and B) of 5 test subjects each. After acclimatising the mice to a feeding room (25°C) with sufficient grain for one week, a nose drop test was conducted. Each mouse received 2–3 nose drops of a pre-suspension fluid ranging from 0.5–1.0 × 10^7^ colony-forming units (cfu) per millilitre depending on the group (i.e., CK, A or B). Initially, the operating personnel were nervous when administering the nose drops to a particularly resistant mouse. The mouse bit an operator, which caused the operator to drop the mouse, resulting in the mouse’s death. Consequently, we decided that the nose drop technique was unsuitable. Instead, the drinking water for the mice was spiked with the *B*. *cereus* S5 strain, and gastric perfusion tests were performed over the next four weeks, except for holidays. Lavage (1 mL/mouse) was performed once a day. The feed and drinking water were replaced every three days, the cages were regularly cleaned and disinfected, and the bedding was regularly replaced. Eighteen oral tests were conducted per mouse, and drinking tests were performed continuously over the four-week study period. Following the animal toxicity test involving the *B*. *cereus* S5 strain, the mice were observed for one month, during which time they were provided with sufficient SPF grain and sterile pure water. All of the mice were healthy during this period. At the end of this period, the mice were killed under anaesthesia, and their bodies were cremated. The entire experimental period spanned approximately three months. To determine the toxicity of the *B*. *cereus* S5 strain through observation, the growth statuses and weights of the mice were measured at regular intervals.

In the CK group, the mice were provided with Millipore ultrapure water and sufficient grain. In groups A and B, the mice were provided with 200 mL ultrapure water spiked with fermentation liquid as drinking water. The preparation method included the incubation of the *B*. *cereus* S5 strain with TSB at 37°C with stirring at 150 rpm for 17 h (SA1) or 5 days (SB1). Centrifugation was performed to obtain the bacterial mass, which was mixed with sterile physiological saline. The amount of bacteria in the drinking water was approximately 1.0–5.0×10^4^ cfu/mL.

The gastric pre-suspension fluids of group A and group B were composed of vegetative cells and the spore biomass, respectively, with their fermentation liquids. The preparation included obtaining the fermented liquid with a bacteria content of approximately 10^9^ cfu/mL through incubation of the *B*. *cereus* S5 strain in TSB medium at 37°C with stirring at 150 rpm for 17 h (SA2) or 5 days (SB2). The liquid was then diluted 100-fold to prepare the pre-suspension fluid over the range of 0.5–1.0 × 10^7^ cfu/mL.

### Virulence genes tests of the Cd-resistance strain *B*. *cereus* S5

*B*. *cereus* may produce many of kinds of toxins, including cereulide and enterotoxin. Cereulide is encoded by the *ces* gene. Enterotoxin includes hemolysin (BL) and nonhemolytic toxin (NHe) and is the product of single genes, such as *bceT*, *cytK*, etc. Hemolysin (BL) consists of a binding factor and two hemolytic-subunits (L1, 38.5KD, and L2, 43.5KD) and is encoded by genes *hblC*, *hblD* and *hblA*. *PlcR* is a pleiotropic regulator found in *B*. *cereus* generally that activates the expression of toxic genes, such as phosphatase c, protease and hemolytic toxins, etc. The Cd resistance of the strain *B*. *cereus* sp. *S5* was tested the genes of *plcR*, *hbl* (a, c and d), *bceT* and *ces*. Virulence genes using primers and its parameters (S1 Table).The specific test method see S1 File.

### Biosorption studies

#### Effect of the activity of the cadmium-tolerant strain of *B*. *cereus* S5 on cadmium salt removal efficiency

The bacterial suspension included new cultures and the cryopreserved (at 4°C for two months) cultures of the *B*. *cereus* S5 strain cultured for 17 h and for 5 days at 37°C with a 10% bacterial load in TSB. The different activities of this bacterial suspension were tested to determine the removal rates and adsorption capacities. When using the wet bacteria, the bacterial sludge was mixed with a small amount of sterile ultrapure water and set aside as the working broth. Half of the samples were dried at 60°C to assess the removal of the different cadmium ion concentrations from the water by the S5 bacterial sludge under drying and dynamic conditions.

#### Cadmium ion removal performances of different forms of the *B*. *cereus* S5 strain

After preparation of the viable biomass by centrifugation of the *B*. *cereus S5* strain cultures in the TSB medium at 37°C for 5 days, bacterial suspensions were prepared via dilution with a small amount of sterile water. The dead *Bacillus* agent was prepared via sterilisation of the live *Bacillus* at 121°C for 15 min in an autoclave; the same dosages were used for the live and dead *Bacillus* agents. The adsorptive removal performance of the cadmium-tolerant S5 strain in the forms of inactivated *Bacillus*, live *Bacillus*, and activated carbon was assessed with different dosages of live biomass (dry weight) and dead biomass (2.78 g/L) associated with solutions of different Cd^2+^ ion concentrations (1, 5, 10, 20, 50, 100, 150, 200, 250 and 300 mg/L) for 1 h. The residual Cd^2+^ concentration in solution was analysed using ICP-MS after sample preparation by centrifugation at 12,500 rpm for 2 min and dilution with ultrapure water. The same process was used to analyse the control samples with different initial cadmium solutions. Here and throughout the rest of this study, the removal rates and biosorptive capacities of the adsorptions were calculated using Eqs (1) and (2), respectively.

#### Identification of the best cadmium ion removal efficiency for assessment of the adsorption law

Adsorption removal experiments involving multiple doses of live *Bacillus* exposed to different cadmium ion concentrations were conducted to identify the best removal performance and to elucidate the adsorption law (the biosorption isotherms and kinetics). The live spore biomass of the *B*. *cereus* S5 strain was obtained via growth in TSB for 5 days, followed by centrifugation. Small amounts of sterilized ultrapure water were added to samples of this biomass to create samples of different concentrations, which were then set aside. The dry weight and the concentration of the treated control solution were measured simultaneously, and each sample was subjected to three parallel experiments. After processing for 60 min and centrifugation for 2 min at 12,500 rpm, the cadmium concentrations of the supernatants were measured via ICP-MS.

#### Biosorption isotherms and kinetics of the *B*. *cereus* S5 strain biomass in the spore state

Two kinetic models—pseudo-first-order [34] and pseudo-second-order [35]—were used to characterise the cadmium adsorption kinetics of the live S5 strain biomass. The integrated forms of the two models are as follows:

$$q_{t}=q_{e}\left[1-\text{exp}\left(-k_{t}\right)\right]$$ (3)

$$q_{t}=t/\left[\left(1/k_{2}q_{e}^{2}\right)+\left(t/q_{e}\right)\right]$$ (4)

where q_e_ is the amount of the cadmium adsorbed at equilibrium (mg/g) and k_1_ (s^-1^) and k_2_ (g mg^-1^ s^-1^) are the rate constants of the pseudo-first- and pseudo-second-order adsorptions, respectively.

The coefficient of determination (R^2^) and the root mean square deviation (RMSD) were used to measure the ability of the models to fit the data accurately. The RMSD was calculated as follows:

$$RMSD=\sqrt{\frac{1}{n}\sum_{i=1}^{n}{\left(q_{im}-q_{ic}\right)}^{2}}$$ (5)

where n is the number of experimental points and q_im_ and q_ic_ are the ith measured and calculated values, respectively. A higher R^2^ value and a lower RMSD value indicate a better fit.

To study the equilibrium time and adsorption kinetics of cadmium ion adsorption by the active *B*. *cereus* S5 spore biomass, batch experiments were performed under optimized conditions (i.e., 1.38 g/L of biomass, an initial cadmium ion concentration of 109 mg L^-1^, 30°C, 100 rpm agitation speed and pH 6.5) for different time intervals.

Three common isotherms were used to describe the adsorption equilibrium: the Langmuir [36], Freundlich [37] and Langmuir-Freundlich isotherms [38].These isotherms are described by Eqs (6), (7) and (8), respectively:

$$q_{e}=K_{L}q_{m}C_{e}/\left(1+K_{L}C_{e}\right)$$ (6)

$$q_{e}=K_{F}C_{e}^{1/n}$$ (7)

$$q_{e}=q_{m}{\left(K_{L}^{'}C_{e}\right)}^{n^{'}}/\left[{\left(1+K_{L}^{'}C_{e}\right)}^{n^{'}}\right]$$ (8)

where C_e_ is the residual at equilibrium (mg L^-1^), q_m_ is the maximum adsorption capacity (mg/g), and K_L_ is the Langmuir adsorption equilibrium constant (L/mg). K_F_ and n are the Freundlich constants, representing the adsorption capacity (L^-1/n^ mg^-(1-n)^ g^-1^) and the adsorption intensity (which varies with the degree of heterogeneity), respectively. K_L_’ and n’ are the Langmuir adsorption equilibrium constant (L mg^-1^) and the adsorption intensity, respectively.

The experiments conducted to study the adsorption isotherms used different initial cadmium concentrations ranging from 1 mg/L to 336 mg/L. The other experimental conditions were as follows: 1.38 g/L live biomass, 28°C, 60 rpm agitation speed, and pH 6.5. For the kinetics experiments, the live biomass of the S5 strain was mixed with synthetic water, and the residual Cd^2+^ concentrations were measured at various time intervals of up to 6 h.

#### Reusability of the spore suspension of the cadmium-tolerant strain biomass of *B*. *cereus* S5

The effect and reusability of the biological adsorption agents involving *B*. *cereus* S5 strain spore suspensions for the treatment of micropolluted water were investigated. After tap water with 25.08 mg/L Cd^2+^ (measured value) and the 3.3 g/L biological adsorption agent featuring the *B*. *cereus S5* strain spore suspension were allowed to settle for 1 h, the residual concentrations of Cd^2+^ in solution were analysed by ICP-MS following centrifugation at 12,500 rpm for 2 min and dilution. To evaluate the reusability, the following procedures were repeated several times. The adsorbent was reused with the same concentration of bacterial suspension by incorporating sterile water obtained from centrifugation at 12,500 rpm for 2 min after desorption with 0.5 mol/L HCl (20 mL, one time) and pure water (10 mL, two times) as the adsorbent in a solution of 20 mg/L Cd^2+^. Each biosorption experiment was conducted with a known quantity of biomass in a 15 mL Cd^2+^ solution for a fixed contact time. The residual concentration of Cd^2+^ in solution was analysed via ICP-MS.

#### Preparation of a fixed-bed column of the cadmium-tolerant bacterium *B*. *cereus* S5 and a control column for the treatment of cadmium-polluted water

The cadmium resistant bacteria of the *B*. *cereus* S5 strain were fixed with K-04 activated carbon from coconut husk (Hainan Star Activated Carbon Co., Ltd., model K-04,10–20 mesh). First, 60 g of sterilized K-04 activated carbon from coconut husk was mixed with the cultured *B*. *cereus* S5 strain in TSB medium at 37°C and 125 rpm for 24 h. After incubation of the mixture of activated carbon with the cadmium-resistant bacteria for 5 days at 30°C, it was packed into glass columns (3.1 cm in diameter) with a fixed bed depth of 21.5 cm and washed with sterile saline.The columns were soaked with 225 mL TSA culture medium for over 2 days to obtain fixed-bed columns (B Column, S5-K04). A control column was prepared by soaking 60 g of sterile K-04 activated carbon from coconut husk in 225 mL TSA culture medium for over 2 days, and this material was then packed into glass columns (3.1 cm in diameter) with a fixed bed depth of 21.5 cm (D Column, CK-K04).

After the fixed-bed column of the cadmium-tolerant bacterium *B*. *cereus* S5 and the control column were prepared for the treatment of cadmium-polluted water, 15.2 mg/L Cd^2+^ cadmium-polluted water was run through the column at a rate of 7 mL/min (the cadmium-polluted water was prepared with raw water from the Panyu Shawan water plant, Guangzhou, Guangdong, with an ammonia nitrogen content of 5 mg/L and a COD of zero). The experiments were conducted at room temperature, and the Cd^2+^ solution was continuously pumped in an upward-flow into the column to avoid channel effects and increase the retention time [39–40]. Samples were collected from the effluent at designated time intervals for the measurement of the residual metal concentrations. The metal biosorption of the fixed-bed column was considered to have reached saturation when no further Cd^2+^ adsorption occurred or when the effluent Cd^2+^ concentration (C) was equal to the influent Cd^2+^ concentration (C_0_), i.e., when the ratio C/C_0_ was approximately 1.0. Meanwhile, when the ratio of C/C_0_ was just above zero, the column was at the breakthrough point or the Cd^2+^ concentration of the influent water was greater than zero. The residual concentration of Cd^2+^ in solution was analysed via ICP-MS. Note that this study did not include a statistical experimental design (e.g., central composite design, confounded factorial design) [41].

### FTIR analysis

The infrared spectra of the bacterial strain before and after metal adsorption and after chemical modification were recorded on a Shima-dzu FTIR spectrometer using a high-sensitivity pyroelectric detector (DLATGS) in the region of 4000–400/cm. The samples were pressed into spectroscopic-quality KBr pellets with a sample/KBr ratio of approximately 1/100. The FTIR spectra were recorded with 500 scans at a resolution of 4 cm^-1^.

### Statistical analysis

Factorial ANOVA was used to test for differences in the removal and adsorption capacities of cadmium ions among treatments. For each treatment, tests were conducted in triplicate, and the mean values were used as the data. The significance of difference was calculated using the statistical software SPSS V 13.0.

## Results

### Isolation of cadmium-tolerant strains from polluted soils

The goal of the preliminary portion of this study was to select and identify the strains with the highest resistance to heavy metal toxicity. Tolerance can be defined as the ability of a microorganism to cope with metal toxicity using its intrinsic properties, whereas resistance is the ability of a microorganism to survive under conditions with high concentrations of toxic metals via detoxification mechanisms that are activated in direct response to the presence of metals. To identify strains that could be used in drinking water treatment, the safety of the strains must be tested.

In this study, the streak plate technique was used to isolate more than 100 strains of cadmium-tolerant bacteria and fungi from cadmium-polluted soil. After the assessment of the cadmium tolerance of the isolates, 17 strains of bacteria (including S5 and S27) and 5 mould strains (including the mould strains S47, S50 and S52) that could resist 10–13 mmol/L of cadmium chloride were further isolated and purified.

### Cadmium ion adsorption testing of the isolates

According to the sensitivity tests involving cadmium salt, the cadmium adsorption abilities of the cadmium-tolerant bacteria and fungi were determined. The performance results for cadmium ion adsorption are shown in Table 1.

**Table 1:** Cadmium removal by the bacterial and the mould strains.

Sample	Cadmium salt removal (%)			
	*C_0_(mg/L)			
	C_1_ (127.7)	C_2_ (318.1)	C_3_ (506.3)	C_4_ (839.8)
**NB**	39.0	29.7	48.4	39.9
**S5**	77.0	83.1	72.1	79.8
**S27**	50.5	68.8	69.2	77.4
**SB**	9.6	10.9	24.9	25.9
**S47**	59.3	68.2	70.8	82.9
**S50**	82.6	85.0	69.6	69.1
**S52**	72.9	69.1	56.4	60.1

Note

*C_0_ (mg/L) refers to the initial concentration of Cd^2+^ (mg/L) in the control.

The removal rates of Cd^2+^ were 29.7%-48.4% and 9.6 to 25.9% for the nutrient broth medium (NB) and the Sabouraud's medium (SB) under different initial Cd^2+^ concentrations (mg/L) for the control, respectively. Among the bacterial strains, the S5 strain removed the highest amount (72.1%-83.1%) of Cd^2+^ (mg/L) from the medium, but for the mould strains, between C1 and C2 concentration of the Cd^2+^ in the medium, the strain S50 showed the best removal up to 82.6 ~ 85%, while between C3 and C4 concentration of the Cd^2+^ in the medium, the strain S47 had the best effect, and the removal rate reached approximately 70.8 to 82.9%.

### Cadmium removal efficiency testing of the tolerant strain suspensions

The cadmium removal efficiencies of the tolerant strain suspensions including the cadmium-resistant bacteria and fungi in the tap water are shown in Table 2.

**Table 2:** Cadmium removal efficiencies of the tolerant strain suspensions.

Sample	Cadmium salt removal (%)		
	*C_0_(mg/L)		
	C_1_ (5.46)	C_2_ (68.4)	C_3_ (323.4)
**S5**	88.7	88.7	83.6
**S27**	74.4	67.1	28.0
**S47**	76.6	57.6	31.6
**S52**	77.1	24.6	17.0

Notes

*C_0_ (mg/L) refers to the initial concentration of Cd^2+^ (mg/L) in the control. The dosage of sample S5 and S27 was all 5.0g/L; The dosage of sample S47 and S52 was all 10.0g/L.

The cadmium removal efficiencies of the tolerant strains suspensions in the tap water were related to the initial Cd^2+^concentrations (mg/L) in the tap water when testing, and the suspensions prepared from the fresh biomass of the selected resistant bacteria had a better absorption rate for the cadmium ions than that of the selected tolerant fungal biomass. The suspension prepared from the fresh biomass of the S5 strain was the best.

All the selected tolerant strains had a high tolerance to cadmium ions, but the active biomass of the S5 strain had the highest capacity for cadmium ion absorption in the tap water, so the S5 strain was chosen to be the object strain.

### Growth curve and morphological observations of the cadmium-tolerant strain S5

**Fig 1:**
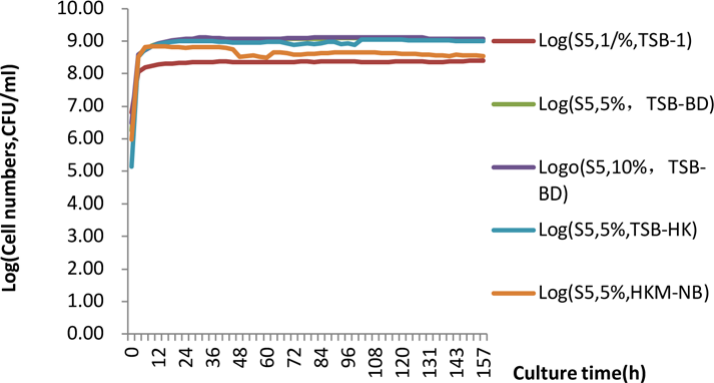
Growth curves of different S5 strain inoculations in different media.

**Fig 2:**
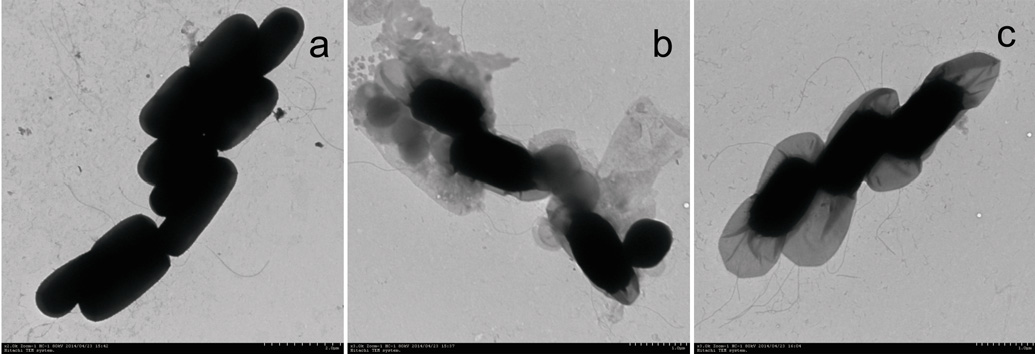
TEM images of the S5 strain cultured on NA medium for different durations (a: 17 h, b: 5 d, and c: 15 d).

Fig 1 shows the growth curves of the S5 strain based on the cultures in TSB (BD) and NB (HK) broth, with 1%, 5% and 10% liquid cultures of the cadmium-tolerant S5 strain (150 mL TSB medium in 500-mL conical bottles, shaken at 150 rpm, at 37°C, for 17 h), respectively. The morphology of the S5 strain cultured under different conditions is shown in Fig 2.

Fig 1 shows that the Bacto^TM^ TSB (BD) medium was the best growth medium, while the HKM-TSB and NB media were also suitable for the S5 bacteria. After culturing for 5 days in TSB medium, the biomass yield of the S5 strain (over the range of 1%-10% inoculation) was not significantly different. The growth curves of the S5 strain in the Bacto^TM^ TSB (BD) medium exhibited several stages. In the first 17–24 h, a maximal increase in the OD_600_ value occurred, and the S5 strain cultured at 37°C was in the exponential growth phase. At 24–60 h, the OD_600_ value dropped slightly. At 60–72 h, the OD_600_ increased again. These findings indicate that the growth of the spores pauses and then resumes.

The bacterium with the highest cadmium tolerance (S5) was examined under a microscope (Hitachi H-7650, 80 kV, 3000×) after being cultured at 37°C in TSB medium for various durations. After being cultured for 17 h, the bacterium was rod-shaped, and the vegetative cells had a diameter of 1.2 μm and a length of 3~4 μm (Fig 2A). Meanwhile, after being cultured for 5 d, spore cores with a diameter of 0.8~0.9 μm and a length of 1.5~1.7 μm were observed (Fig 2B).Finally, after being cultured for 15 d, the spores had a diameter of 0.8~0.9 μm and a length of 1.3~1.7 μm and began to lyse (Fig 2C). The vegetative cells had rounded ends and occurred singly or in pairs. Flagella could be observed in the vegetative cells and spore stage. The bacteria were Gram-positive in both the exponential and stationary growth phases.

The TEM images of the S5 strain at different incubation times clearly indicate that the formed spores were relatively full and active after culturing in TSB liquid medium for 5 days. However, after 15 days, the spores began to lyse, and their vitality rapidly decreased.

### Identification of the cadmium-tolerant strain and its cadmium tolerance genes

**Fig 3:**
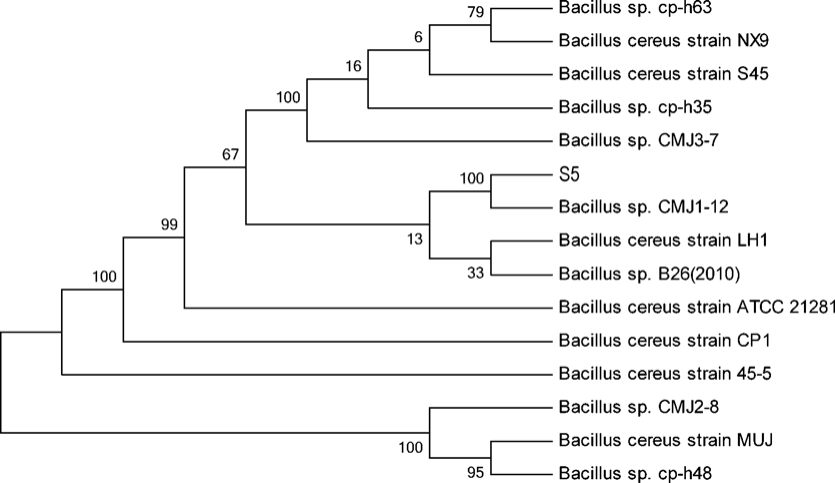
The maximum parsimony tree of the S5 strain.

We identified the cadmium-tolerant strain by amplifying a partial sequence of the 16S rDNA gene (GenBank accession number: KU927490)from the cadmium-tolerant strain of S5. Based on the 16S rDNA gene sequences from GenBank releases with high similarity (>99%), we observed that this strain, which had good tolerance levels and effectively removed Cd2+ from solution, was most closely genetically related to B. cereus sp. Cp1. The maximum parsimony tree of the S5 strain is shown in Fig 3. According to multiple analysis results, we placed the cadmium-tolerant strain of S5 in the species of B. cereus S5.

**Fig 4:**
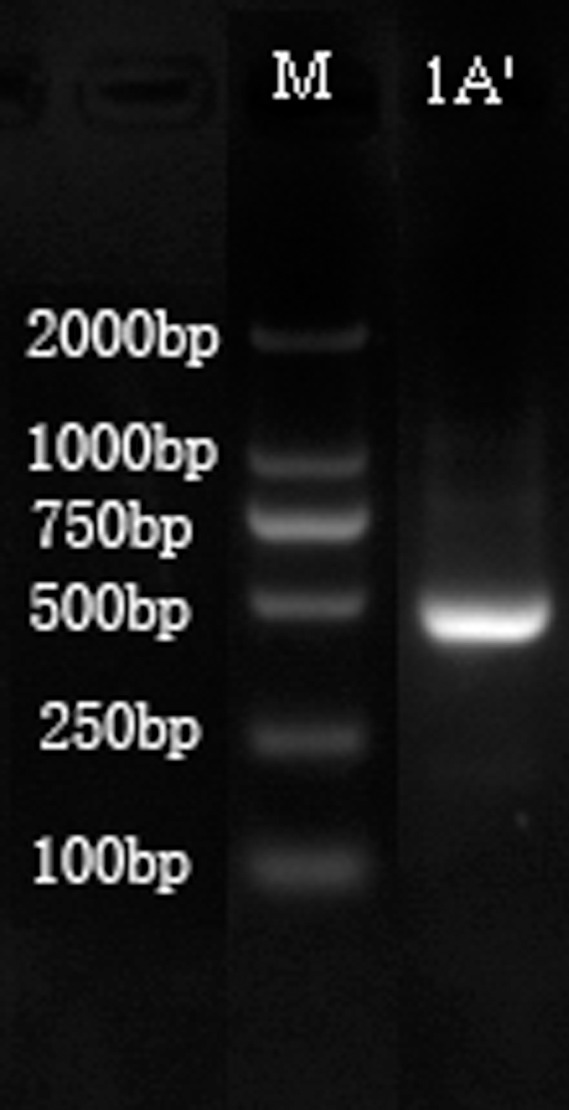
Nested PCR results of cadmium ion tolerance genes in the strain of *B*. *cereus* S5 (Lane 1, DL 2000 DNA Marker, TakaRa; Lane 2, the second round of PCR amplification).

After the cadmium-tolerant strain was identified as the species *B*. *cereus* sp. *S5*, the cadmium tolerance genes of the *B*. *cereus* S5 strain were identified further. A *Cad* A cationic drain system has been found in Gram-positive bacteria and is associated with ATPase. Therefore, the ATPase genes in the Cd-resistant strain *B*. *cereus* S5 were investigated using nested PCR technology. The agarose gel electrophoresis of the cadmium tolerance genes are shown in Fig 4.

No bands were found in the agarose gel electrophoresis of the first round of nested PCR. However, a band of 450 bp was revealed by sequencing the cutting purification material of the second round of PCR amplification products (GenBank accession number: KU519618), and the similarity of the cadA sequence of the Cd-resistant strain *B*. *cereus* S5 with that of *Bacillus* sp. N11 and *Bacillus firmus* was 84% via BLAST using the NCBI database. The results of the second step of the nested PCR revealed a resistance system *cad*ABC with p-type ATPase in the Cd-resistant strain *B*. *cereus S5*. The resistance function coded by the cadA determinant results from decreased intracellular accumulation of Cd^2+^, mediated by an energy-dependent efflux mechanism [42].

### Preliminary toxicity testing of the cadmium-tolerant strain of *B*. *cereus S5*

After nearly a month of feeding and drinking tests, all of the mice were alive and healthy, except for the mouse from group B that was accidentally sacrificed. The genetic factors of the second group and the weights of the mice orally administered S5 bacteria by direct gavage for nearly one month are shown in S2 Table.

The results showed that the viable biomass of the *B*. *cereus* S5 strain and its broth were not only non-toxic to the mice but also resulted in weight gain, indicating that the mice were healthy. This preliminary toxicity evaluation provides strong support for the security of the cadmium-tolerant strain *B*. *cereus* S5 isolated from the contaminated points.

After a month of toxicity testing, the small, white mice receiving doses less than 10^7^ cfu/g remained alive and healthy, but because the cadmium-tolerant strain *B*. *cereus* S5 is still a less toxic strain of conditional pathogenic bacteria (S1 Fig), it is not suitable for addition to drinking water. However, this strain can feasibly be applied to the water during front-end processing in a bioremediation reactor because a variety of joint processing technologies and water disinfection processes occur before the water enters the network.

### Biosorption studies

#### Cadmium salt removal efficiencies of the *B*. *cereus* S5 strain suspensions with different activity levels

The cadmium salt removal results of the *B*. *cereus* S5 strain suspensions with different activity levels are shown in Table 3.

**Table 3:** Cadmium removal efficiencies of *B*. *cereus* S5 strain suspensions with different activity levels.

Sample	Cadmium ion removal (%)			Cadmium ion adsorption (mg/g)		
*C_0_ (mg/L)	C_1_ (9.1)	C_2_ (70.1)	C_3_ (254.0)	C_1_ (9.1)	C_2_ (70.1)	C_3_ (254.0)
**S5-A1**	73.1	64.6	57.4	0.8	5.7	18.2
**S5-A2**	95.5	90.6	87.5	1.1	7.9	27.8
**S5-B1**	71.8	75.0	56.5	0.8	6.4	17.5
**S5-B2**	94.4	89.4	81.0	1.0	7.6	25.1
**S5-C1**	62.5	65.8	60.5	0.6	4.9	16.5
**S5-C2**	82.3	67.9	66.9	0.8	5.1	18.3

Notes: 1) *C_0_ (mg/L) refers to the initial concentration of Cd^2+^ (mg/L) in the control; 2) S5-A1 is the dry bacterial powder obtained after the *B*. *cereus* S5 strain was cultured in TSB for 5 days and dried at 60°C; 3) S5-A2 is the wet bacterial sludge obtained after the *B*. *cereus* S5 strain was cultured in TSB for 5 days and centrifuged; 4) S5-B1 is the dry bacterial powder obtained after the *B*. *cereus* S5 strain was cultured in TSB for 5 days, stored at 4°C for two months, and dried at 60°C; 5) S5-B2 is the wet bacterial sludge obtained after the *B*. *cereus* S5 strain was cultured in TSB for 5 days, centrifuged, and stored at 4°C for two months; 6) S5-C1 is the dry bacterial powder obtained after the *B*. *cereus* S5strain was cultured in TSB for 17 h and dried at 60°C; 7) S5-C2 is the wet bacterial sludge obtained after the *B*. *cereus* S5 strain was cultured in TSB for 17 h and centrifuged; 8) The concentration of the biological adsorbent was 8.3 g/L, which was used to convert the values to dry weights.

The results demonstrate that the wet bacterial sludge of the *B*. *cereus* S5 strain (S5-A2) cultured for 5 days had the highest cadmium-removal efficiency. The fresh spores had a higher adsorption capacity than the previously frozen spores (the results show that the spore vitality was slightly affected by storage in the wet state at 4°C for two months). The cadmium removal performance of the biomass varied under drying and dynamic conditions, and the cadmium absorption capacity of the *B*. *cereus* S5 bacterial sludge was higher under high cadmium concentrations.

#### Cadmium ion removal performances of different forms of the *B*. *cereus* S5 strain

**Fig 5:**
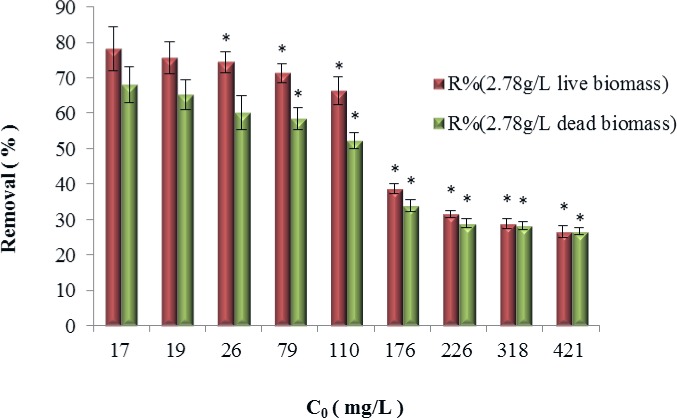
Removal efficiency of live and dead *B*. *cereus* S5 strain biomasses. (*p<0.05,*based on the value of 17mg/LCd^2+^, p value refers to the significant differences between the bars.)

**Fig 6:**
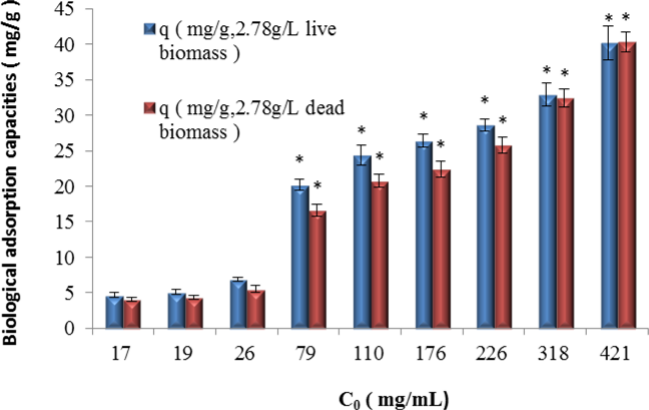
Biological adsorption capacities of the live and dead of the *B*. *cereus* S5 strain biomasses (*p<0.05,*based on the value of 17mg/LCd^2+^).

The cadmium ion adsorptive removal performances of different forms of Cd^2+^-tolerant bacteria of the *B*. *cereus* S5 strain are shown in Figs 5 and 6, which show the results for the live and dead biomasses of the Cd^2+^-tolerant *B*. *cereus* S5strain.

The results show that the live and dead biomasses of the Cd^2+^-tolerant *B*. *cereus* S5 strain were able to adsorb cadmium ions in solution but that the live biomass outperformed the dead biomass at lower Cd^2+^ concentrations (<226 mg/L). However, when the Cd^2+^ concentration exceeded 318 mg/L, the removal performances of both types of biomass were nearly the same.

Metal bioremediation by growing cells is particularly promising because some bacterial strains possess high tolerance to various metals and are thus potential candidates for the simultaneous removal of numerous metals from wastes. Furthermore, the ability of growing cells to degrade metal complexes and remove other organic/inorganic contaminants present in waste may assure that the effluent is suitable for environmental discharge [43].

#### Identification of the best cadmium ion removal efficiency for assessment of the adsorption law

We determined that the performance of the active *B*. *cereus* S5bacterial spore biomass as a biological adsorbent in terms of removing cadmium ions from solution is affected by various factors, including the absorbent dosage, cadmium concentration and treatment time. Under certain processing times, the cadmium ion removal performance was related only to the adsorbent dosage and cadmium ion concentration.

**Fig 7:**
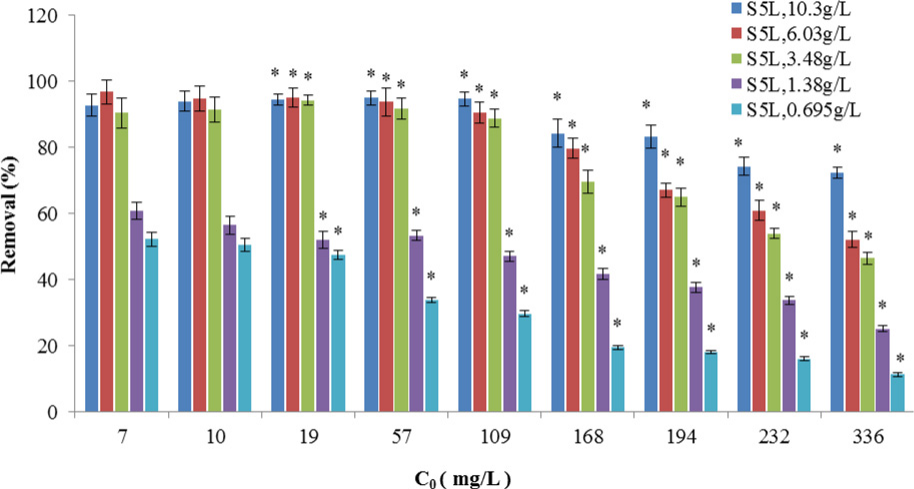
Removal efficiency of the S5L strain under different Cd^2+^ concentrations (mg/L) (*p<0.05,*based on the value of 7mg/LCd^2+^).

**Fig 8:**
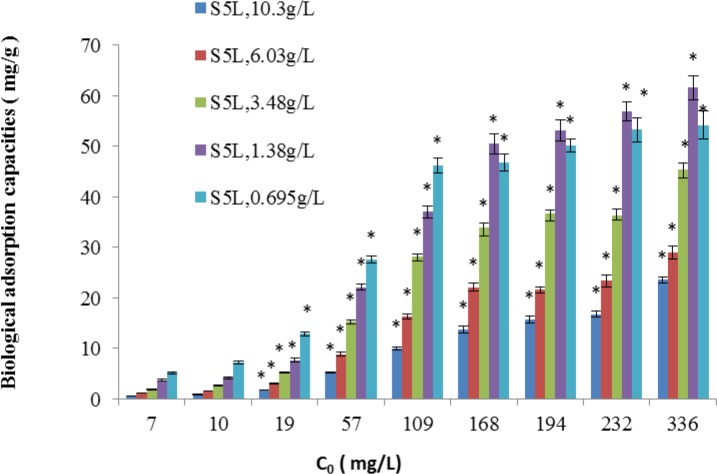
Biological adsorption capacity (mg/g) of the S5L strain under different Cd^2+^ concentrations (mg/L, *p<0.05,*based on the value of 7mg/LCd^2+^).

The removal efficiency and biological adsorption of the live *B*. *cereus* S5biomass (S5L) under different Cd^2+^ concentrations (mg/L) are shown in Figs 7 and 8, respectively.

The removal performance of the bacterial suspensions of the *B*. *cereus* S5active spores for different cadmium concentrations was related to the dosage of the*B*. *cereus* S5spore suspension. For Cd^2+^ concentrations (mg/L) of 1–109 mg/L, approximately 3.48–10.3 g/L of active *B*. *cereus* S5spore biomass resulted in good removal efficiency (>80%, Fig 7). For a Cd^2+^ concentration of 336 mg/L (mg/L), an active *B*. *cereus* S5 spore biomass dosage of 1.38 g/L demonstrated a maximum biological adsorption capacity of 61.53 mg/g, but its removal efficiency was only approximately 30%.

The selected adsorbent dosage was crucial to the adsorption process because a larger dosage provides more active sites but increases unsaturated adsorption. The initial concentration can strongly affect the adsorption kinetics. At a high initial concentration, the gradient between the solution and the adsorbent enhances the Cd^2+^ adsorption because the ions surround the adsorbent surface and occupy the vacant spaces in the biomass. The adsorption capacity is expected to increase with the initial Cd^2+^ solution concentration until reaching equilibrium at a certain initial concentration, and our experimental results confirm this behaviour. According to the results and the goal of this study, which was to provide a means of remediating water, the adsorption studies of the isotherms and kinetics of the *B*. *cereus* S5 strain biomass in the spore state were conducted using the following experimental conditions: temperature of 30°C, 100 rpm mixing, initial pH of 6.5, active *B*. *cereus* S5 spore biomass dosage of 1.38 g/L, and initial Cd^2+^ concentration of 109 mg/L.

#### Adsorption isotherms and kinetics of the *B*. *cereus* S5 strain biomass in the spore state

**Fig 9:**
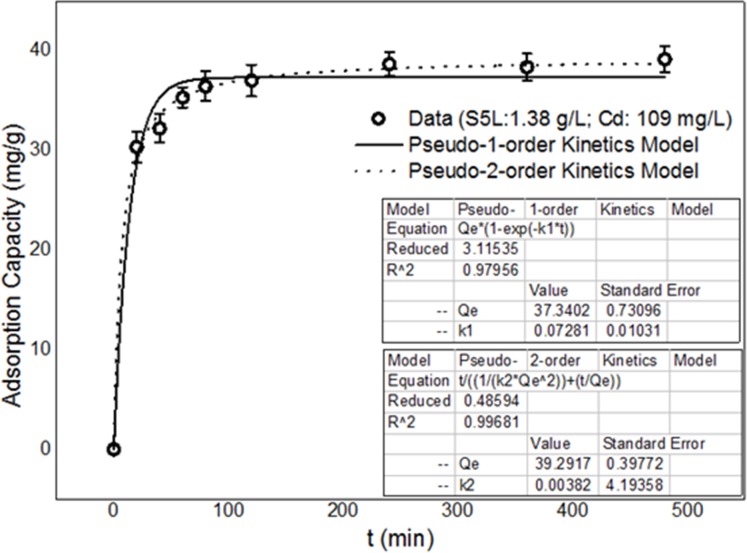
Fitting of the adsorption data for S5L by kinetic models.

**Fig 10:**
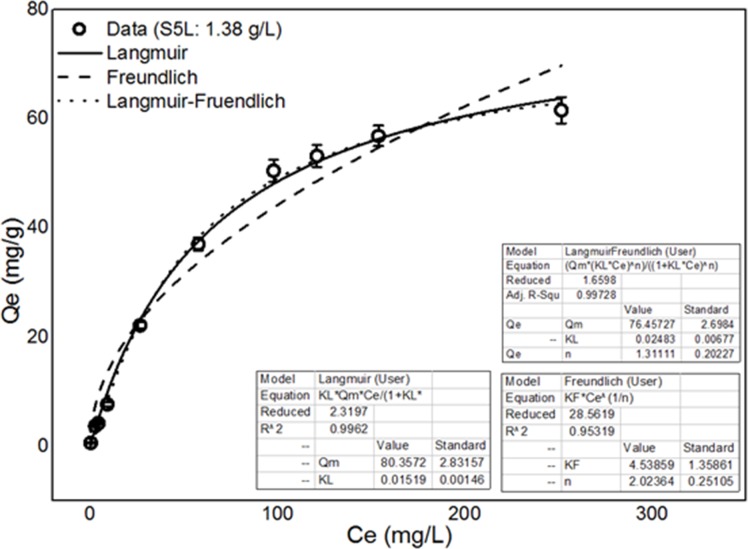
Fitting of the adsorption data for S5L to isotherm models.

Figs 9 and 10 show the biosorption kinetics and isotherms, respectively, for the *B*. *cereus* S5strain biomass in the spore state (S5L) as a biological adsorbent.

Table 4 shows the results of the kinetic and isotherm models fitting to the cadmium adsorption data for S5L.

**Table 4:** Kinetic and isotherm models used to fit the cadmium adsorption data for S5L.

Model	Parameter	Value
	Kinetic models	
**pseudo-first order**	q_e_(mg g^-1^)	67.57
	K_1_ (min^-1^)	0.08302
	R^2^	0.991
	RMSD	1.9291
**pseudo-second order**	q_e_ (mg g^-1^)	70.16
	K_2_ (min^-1^)	0.00293
	R^2^	0.997
	RMSD	1.2114
	Isotherm models	
**Langmuir**	K_L_ (L mg^-1^)	0.01519
	q_m_ (mg g^-1^)	80.357
	R^2^	0.9962
	RMSD	1.3623
**Freundlich**	K_F_ (L^-1/n^ mg^-(1-1/n^) g^-1^)	4.5386
	N	1.3111
	R^2^	0.9532
	RMSD	4.78012
**Langmuir-Freundlich**	KL’ (L mg^-1^)	0.02483
	N’	2.024
	Qm (mg g^-1^)	76.46
	R^2^	0.9973
	RMSD	1.0779

Based on the R^2^ values (all greater than 0.95) shown in Table 3, the above three models fit the experimental data for the live biomass well, implying that the biosorption of Cd^2+^ ions is a complex process involving more than one surface binding mechanism (Fig 9). However, the R^2^ values indicate that the Langmuir and Langmuir-Freundlich isotherm adsorption models fit the cadmium ion adsorption data best. This finding predicts a cooperative adsorption process involving adsorbent-adsorption interactions [44–45]. The results also show that the pseudo-second- and pseudo-first-order kinetic models fit the adsorption data well, with q_e_ values of 70.16 and 67.57 mg/g and R^2^ values of 0.997 and 0.991, respectively (Fig 10). However, the R^2^ and RMSD values in Table 3 indicate that the pseudo-second-order model was more appropriate. Thus, the adsorption process was likely controlled by a chemical adsorption mechanism.

#### Reusability of the spore suspension of the cadmium-tolerant strain biomass of B. cereus sp. S5

The biological adsorption agent for treating Cd^2+^-polluted water showed a good removal rate (>78%) after five reuses when the dosage was 3.3 g/L and the initial Cd^2+^ concentration was 25.08 mg/L. The adsorption capacities of the first three cycles were 9.73, 9.30, and 8.27 mg/g, respectively. These results suggest that *B*. *cereus* S5strain biomass in the spore state could be used at least three times to treat micropolluted water because the agent retained more than 85% of its maximum adsorption capacity.

After the heavy metal ions were absorbed by microbial cells, the degree of heavy metal desorption by the microorganisms needed to be decreased to prevent secondary pollution and promote recovery of the metal. Several methods are available to limit desorption of heavy metals from microbial cells. Commonly used desorption agents include hydrochloric acid, sulphuric acid, nitric acid, acetic acid, thiourea, carbonate and EDTA. In a previous study, when *Rhizopus nigricans* was exposed to 0.5 mol/L HCl following the adsorption of a certain amount of heavy metals, the desorption rates of Pb^2+^, Cu^2+^, Mn^2+^ and Cr^6+^ were 88%, 67%, 75% and 60%, respectively [46].

This study did not examine heavy metal desorption in depth, and only simple repeated washing was conducted. Thus, additional detailed studies are required in this area to ensure the maximum adsorption capacity of the biological materials and to reduce the costs of heavy metal pollution treatment.

#### Effect of the immobilisation of *B*. *cereus* S5 cadmium-tolerant bacteria through activated carbon fixation for the treatment of cadmium-polluted water

**Fig 11:**
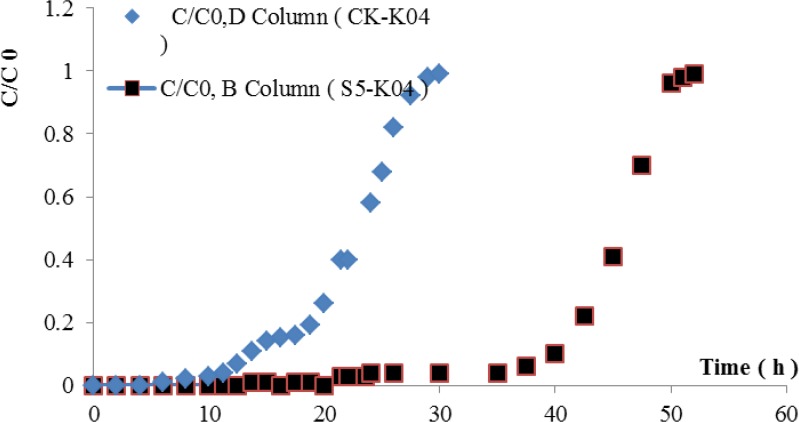
Breakthrough curve profiles of the S5-C04 and CK-C04 adsorption columns (C_0_ of 15.2 mg/L Cd^2+^, flow of 7.0 mL/min).

In this study, an immobilized reaction column fabricated by the activated carbon fixation of cadmium tolerant bacteria (B Column, S5-K04) and a control column of saturated active carbon made from the nutrient salt used in cultivation of the bacteria (D Column, CK-C04) were investigated.Cadmium-contaminated water was run through these columns at a flow rate of 7 mL/min for nearly 3 days using two creeping pumps. The cadmium ion concentrations detected in the effluent at different sampling times are shown in Fig 11.

When the immobilised reaction column (B Column, S5-K04) was run with 15.2 mg/L Cd^2+^ cadmium-polluted water at a flow rate of 7 mL/min, the breakthrough time was approximately 40 h, and the saturated adsorption time was approximately 50 h. In contrast, for the control column run under the same conditions, the breakthrough time was approximately 10 h, and the saturated adsorption time was approximately 30 h. The results show that the cadmium-tolerant bacteria activated carbon immobilised column could be used for a longer period and provide more effective treatment of cadmium-polluted water than the control column. Thus, the*B*. *cereus* S5 Cd-resistant strains with high endurance can be used for the treatment, both emergency and routine, of cadmium-polluted water.

Few reports describe cadmium-resistant bacteria biomass with the ability to remove cadmium ions from raw water. In this paper, the removal of cadmium from cadmium-polluted water prepared using raw water was conducted. During the continuous running experiment, after running a cycle followed by elution to cadmium desorption and re-culturing, the reaction column of immobilised bacteria could still be used for two additional rounds in the experiment with very good effect (data not provided). This finding proves that cadmium-resistant bacteria of the *B*. *cereus* S5strain in the column were still active (the author deemed that further study of the activity of cadmium-tolerant microorganisms during the removal of cadmium ions at different concentrations is needed). This study utilized an immobilization material consisting of activated carbon with a very good adsorption efficiency fixed with high-cadmium-tolerant *Bacillus* strains screened from Cd-contaminated soil to remove cadmium ions from water polluted with 15~25 mg/L Cd^2+^ ions and a small amount of ammonia nitrogen. After 55 h of continuous operation, the fixed strain was still alive, which is related to the performance of the strain, including its high Cd^2+^ resistance and spore forming ability. In this study, an initial concentration of 15~25 mg/L Cd^2+^ of the contaminated water was used, which has the advantage of allowing a fast response to the immobilised reaction column. However, the cadmium ion concentration in actual raw water usually does not exceed 10 times the environmental water quality standard, which is 0.5 mg/L. The useful lifetime of the immobilised reactor in this study was much higher than the results of previous studies. Therefore, the cadmium-tolerant strain of *B*. *cereus* S5 is promising for practical applications in the removal of cadmium ions from actual micro-polluted water or in the emergency treatment of water.

### FTIR analysis

To understand the intrinsic factors involved in the adsorption effect of cadmium ions by the microbial strain *B*. *cereus* sp. *S5*, infrared spectrum analysis was carried out before and after the activity of *Bacillus* biomass and the inactivated spore biomass adsorption of cadmium ions.

**Fig 12:**
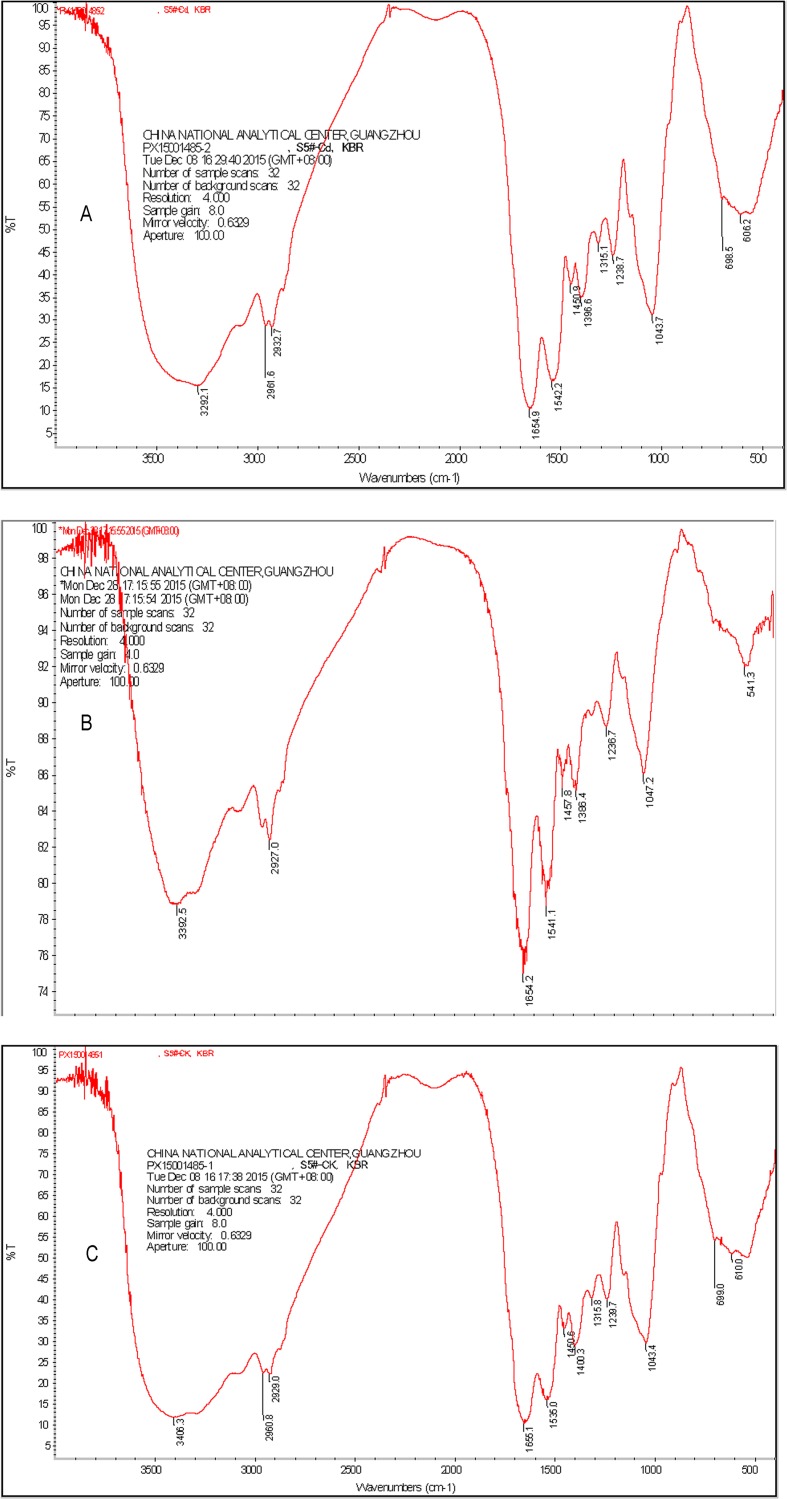
The infrared spectra of the activity of *Bacillus* biomass and the inactivated spore biomass adsorption of cadmium ions. Curve A is the FTIR spectrum of the activity of *Bacillus* biomass adsorption of cadmium ions, curve B is the FTIR spectrum of the inactivated spore biomass adsorption of cadmium ions, and curve C is the control curve (the activity of *Bacillus* biomass with no adsorption of cadmium ions).

The FTIR spectra (Fig 12) reveal the presence of amino, carboxyl and hydroxyl groups on the pristine biomass. The strong bands in the region 3600–3000/cm are characteristic of N–H and O–H stretching vibrations. The peak positions at 1043.4 and 1239.7/cm are assigned to alcoholic C–O and C–N stretching vibrations, respectively, suggesting the presence of hydroxyl and amine groups on the biomass surface. The bands at 2960.8 and 2929.0/cm exhibit the C–H stretching vibrations of alkyl chains attributed to fatty acids found in membrane phospholipids, and the band at 1535.0 suggests the presence of an amido bond.

The amide I band primarily reflects C–O stretching vibrations and is centred at 1655.1/cm, whereas the amide II band is a combination of N–H bending and C–N stretching at approximately 1535.0/cm and is characteristic of the IR absorption of protein, which could represent one of the significant components of the cell wall. The wave number at 1400.3/cm can be attributed to the COO^-^ of the carboxylate group present in the biomass.

The FTIR bands of Cd ^2+^ adsorption (Fig 12, curves A and B) revealed that the peaks at 1655.1 and 1535.0/cm attributed to the amide (I and II) bands had shifted to 1654.2 and 1541.1/cm, respectively, following Cd^2+^ adsorption, suggesting that the nitrogen atom may be the main adsorption site for Cd^2+^ attachment on the activity of *Bacillus* biomass. In addition, the broadening and stretching of the band characterising the presence of C–O at the range of 900–1380/cm indicates the interaction of the metal with C–O groups on the surface of the biomass. Changes in the FTIR spectrum at wave numbers 3406.3–3284.4/cm and 1043.4–1047.2/cm are attributed to the stretching vibrations of the alcohol groups and C–O stretching vibrations, respectively. Another significant change upon Cd^2+^ adsorption is the splitting of the peak at 3406.3/cm into 3392.5 and 3284.4/cm. This region may correspond to both C–H and O–H stretching. These changes suggest the possibility that the oxygen atoms in the hydroxyl groups of the activity of *Bacillus* biomass are involved in this adsorption process as well. The transmittance of the peaks in the loaded biomass is substantially lower than those in the pristine biomass. This indicates that bond stretching occurs to a lesser degree due to presence of metals and that the following peak transmittance is reduced. These results are in agreement with those of Oh et al. [47–48].

## Discussion

*Bacillus cereus* sp. *S5*, which was screened from cadmium-contaminated soil in this study, is a gram-positive, cadmium-resistant bacterium. This strain has a high tolerance to cadmium ions, and the active spore biomass of *B*. *cereus* S5has a high capacity for cadmium ion adsorption. Relative to the cadmium-resistant fungi [49], the active biomass of *B*. *cereus* S5 strain has a higher absorption rate in water, and relative to the tolerant bacteria of *Klebsiella* sp. from other via screening to cadmium [50], it grows more rapidly. When the *B*. *cereus* S5strain was cultured in nutrient containing cadmium ions, the amount of cadmium ions in the nutrient solution was reduced after centrifugation. The results suggest that the resistant bacterium may not participate in the degradation of cadmium but rather the adsorption.

The analysis of the cadmium tolerance genes of *B*. *cereus* S5 revealed the presence of ATPase genes that are associated with cadmium tolerance and that indicate a pumping mechanism of ATP. The FTIR spectra revealed the presence of amino, carboxyl and hydroxyl groups on the pristine biomass and indicated that the cadmium ion removal ability was related to the structure of the strain.

## Conclusions

In this study, we have identified a new bacterial strain, classified as *B*. *cereus S5*. This strain exhibited high Cd resistance and effective Cd removal from cadmium-polluted water. Both the inactivated and activated forms of the Cd-resistant strain of *B*. *cereus sp*.*S5* removed Cd^2+^ well, with good cadmium ion absorption capacities. Furthermore, its active spores exhibited a good adsorption capacity for cadmium ions, exceeding the capacities of the aforementioned inactivated and activated forms. However, the live biomass of the *B*. *cereus* S5 strain outperformed the dead biomass at lower Cd^2+^ concentrations. The Langmuir and Langmuir-Freundlich isotherm adsorption models were able to fit the cadmium ion adsorption data, and the adsorption curves revealed second-order reaction kinetics. For Cd^2+^ concentrations (mg/L) of 1–109 mg/L, active spore biomass of the *B*. *cereus* S5 strain at a dosage of approximately 3.48–10.3 g/L provided good removal efficiency (>80%). Furthermore, the cadmium-tolerant bacteria-activated carbon-immobilised column could be used for longer and was more effective in the treatment of cadmium-polluted water than the control column. In addition, a toxicity safety test using mice demonstrated that the biomass of the isolated *B*. *cereus* S5 strain and its fermentation products were non-toxic.

Thus, the isolated *B*. *cereus* S5 strain can be considered an alternative biological adsorbent for cadmium-pollution water-treatment techniques aimed at emergency responses to severe cadmium pollution and routine treatment of trace cadmium pollution. However, because the cadmium-tolerant strain B. cereus S5 is a less toxic strain of conditional pathogenic bacteria, which should perform strict control of the water before it enters to the network when this strain applied for cadmium removal from cadmium-polluted water.

## Supporting Information

S1 Fig The PCR figure of the virulence genes tests of the Cd-resistance strain *B*. *cereus* S5. (TIF)

S1 File Preliminary toxicity test of the *B*. *cereus* S5 strain. (DOCX)

S1 Table Virulence genes tested using primers and their parameters. (DOCX)

S2 Table Body weights of mice after direct gavage and drinking of the *B*. *cereus* S5 strain (unit: g). (DOCX)
