# Supplementary figures and images for: Cd-Resistant Strains of B. cereus S5 with Endurance Capacity and Their Capacities for Cadmium Removal from Cadmium-Polluted Water
Source: PLoS One. 2016 Apr 14;11(4):e0151479. doi: 10.1371/journal.pone.0151479 (PMC4831789; doi:10.1371/journal.pone.0151479)

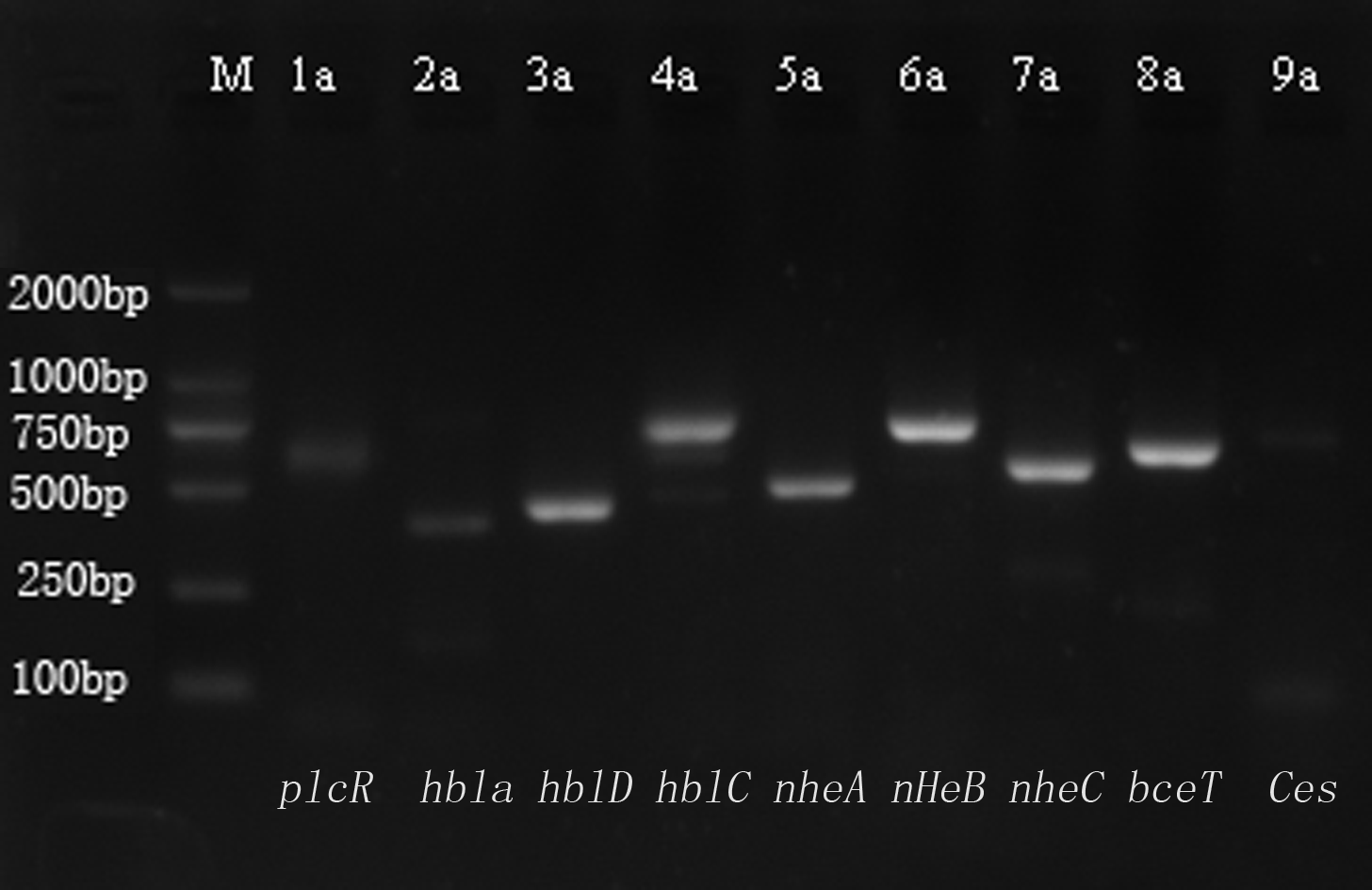

Supplement: S1 Fig — (TIF) [file pone.0151479.s001.tif]
