# Supplementary material for: Cd-Resistant Strains of B. cereus S5 with Endurance Capacity and Their Capacities for Cadmium Removal from Cadmium-Polluted Water
Source: PLoS One. 2016 Apr 14;11(4):e0151479. doi: 10.1371/journal.pone.0151479 (PMC4831789; doi:10.1371/journal.pone.0151479)
Supplement: S1 File — (DOCX) [file pone.0151479.s002.docx]

Cd-resistant Strains of *B. cereus* S5 with Endurance Capacity and Their Capacities for Cadmium Removal from Cadmium-polluted Water

Huiqing Wu^1,2^, Qingping Wu^1,2^*, Guojie Wu^3^, Qihui Gu^1,2^, Linting Wei^1,2^

^1^ State Key Laboratory of Applied Microbiology Southern China, Guangdong Institute of Microbiology, Guangzhou, Guangdong, China

^2^ Guangdong Provincial Key Laboratory of Microbial Culture Collection and Application, Guangdong Open Laboratory of Applied Microbiology, Guangzhou, Guangdong, China

^3^College of  Chemistry and Chemical Engineering, Zhongkai University of Agriculture and Engineering, Guangzhou, Guangdong, China

* Corresponding author

E-mail: wuqp203@163.com

S1 text: The preliminary toxicity test of the *B. cereus* S5 strain

**Materials and methods**

**The animal toxicity test involving the *B. cereus* S5 strain**

The test subjects were 15 male Chinese Kun Ming (KM) specific-pathogen-free (SPF) mice that were 20-25 days old and weighed 18-22 g (Guangdong Province Medical Animal Experimental Centre, SCXK2013-0002). All of the animal procedures complied with the guide for the care and use of laboratory animals and were approved by the Animal Care Committee of the Centre for Disease Control and Prevention of Guangdong Province (Approval ID: 13827183491). The mice were randomly divided into 3 groups, namely the CK, A and B groups, with 5 test subjects in each group. After acclimatizing the mice to a feeding room (25 °C) with sufficient grain for one week, a nose drop test was conducted. Each mouse received 2-3 nose drops of the pre-suspension fluid ranging from 0.5-1.0× 10^7^ colony-forming units (cfu)/mL depending on the group (i.e., CK, A or B). Initially, the operating personnel were nervous when administering the nose drops to a particularly resistant mouse. The mouse bit an operator, which caused the operator to drop the mouse, resulting in the mouse’s death. Consequently, we decided that the nose-drop technique was unsuitable. Instead, the drinking water was spiked with the *B. cereus S5* strain, and gastric perfusion tests were performed over the next four weeks, except for holidays. Lavage (1 mL/mouse) was performed once a day. The feed and drinking water were replaced every three days, the cages were regularly cleaned and disinfected, and the bedding was regularly replaced. Eighteen oral tests were conducted for every mouse, and drinking tests were performed continuously over the four-week study period. Following the animal toxicity test involving the *Bacillus cereus S5* strain, the mice were observed for one month, during which time they were provided with sufficient SPF grain and sterile pure water. All of the mice were healthy during this period. At the end of this period, the mice were killed under anaesthesia, and their bodies were cremated. The entire experimental period was approximately three months. To determine the toxicity of the *B. cereus S5* strain through observation, the growth statuses and weights of the mice were measured at regular intervals.

In the CK group, the mice were provided with Millipore ultrapure water and sufficient grain. In groups A and B, the mice were provided with 200 mL ultrapure water spiked with fermentation liquid as drinking water. The preparation method included the incubation of the *B. cereus S5* strain with TSB at 37 °C with stirring at 150 rpm for 17 h (SA1) or 5 days (SB1). Centrifugation was performed to obtain the bacterial mass, which was mixed with sterile physiological saline. The amount of bacteria in the drinking water was approximately1.0-5.0 × 10^4^ cfu/mL.

The gastric pre-suspension fluids of group A and group B were composed of vegetative cells and the spore biomass, respectively, with their fermentation liquids. The preparation included obtaining the fermented liquid with a bacterial content of approximately 10^9^ cfu/mL through incubation of *B. cereus S5* the strain in TSB medium at 37 °C with stirring at 150 rpm for 17 h (SA2) or 5 days (SB2). The liquid was then diluted 100-fold to prepare the pre-suspension fluid over the range of 0.5-1.0 × 10^7^ cfu/mL.

**Virulence genes tests of the Cd-resistance strain *B. cereus* S5**

*B. cereus* may produce many of kinds of toxins, including cereulide and enterotoxin. Cereulide is encoded by the *ces* gene. Enterotoxin includes hemolysin (BL) and nonhemolytic toxin (NHe) and is the product of single genes, such as *bceT, cytK*, etc. Hemolysin (BL) consists of a binding factor and two hemolytic-subunits (L1, 38.5KD, and L2, 43.5KD) and is encoded by genes *hblC*, *hblD* and *hblA*. *PlcR* is a pleiotropic regulator found in *B. cereus* generally that activates the expression of toxic genes, such as phosphatase c, protease and hemolytic toxins, etc. The Cd resistance of the strain *B. cereus S5* was tested the genes of *plcR*, *hbl* (a, c and d), *bceT* and *ces.* After cultivation for 24 h at 37 °C on tryptic soy agar (TSA), a single colony of pure isolate was inoculated into 10 mL of TSB at 37 °C for 24 h. Genomic DNA of the cadmium-tolerant strains *B. cereus* *S5* was extracted using a genomic DNA extraction kit ( Dongsheng Biotech, Guangzhou, China) according to the manufacturer’s instructions. The concentration of genomic DNA was determined at 260 nm using a Biospec-nano 230V UV spectrophotometer (Shimadzu Corporation, Nishinokyo Kuwabara-cho, Nakagyo-ku, Kyoto 604-8511, Japan). S1Table lists the primers of the virulence genes used in the reactions and its parameters.

The 2× PCR system included a total volume of 25 μL (1 μL each of the primers, 9.5 μL of DDH_2_O, 1 μL of DNA and 12.5 μL of 2× PCR Smarter Mix) and a PCR tube. The amplification reactions were performed in a Bio-Rad United PCR apparatus. The PCR amplification procedure involved the following reaction conditions: pre-degeneration at 94 °C for 3 min; denaturation at 94 °C for 1 min; annealing at 55 °C or 58 °C for 1 min for 30 cycles; extension at 72 °C for 7 min; and finally, preservation at 4 °C.

The amplification products were analysed by gel electrophoresis using 1.5% agarose in 1x TAE buffer with goldview dye in the electrophoresis apparatus EPS 300 ( Shanghai, Tanon; Shanghai, China). Detection was done using the BioDoc Analyze video documentation system (GE Image Quant 350, GE Healthcare, USA).

**Results**

**The animal toxicity test involving the B. cereus S5 strain**

After nearly a month of feeding and drinking tests, all of the mice were alive and healthy, except for the mouse from group B that was accidentally killed. The genetic factors of the second group and the weights of the mice orally administered *B. cereus* S5 bacteria by direct gavage for nearly one month are shown in S2 Table.

The results showed that the live biomass of the *B. cereus* S5 strain and its broth were not only non-toxic to mice but also resulted in weight gain, indicating that the mice were healthy.

**Virulence genes tests of the Cd-resistance strain *B. cereus* S5**

The test results showed the strain had the genes *nheA, nheB, nheC* and *bceT* but no *ces*, and the levels of *plcR* and *hbla* were very weak (S1 Fig). On the basis of inference, the strain is considered a weakly toxic strain.
