# Supplementary material for: Cd-Resistant Strains of B. cereus S5 with Endurance Capacity and Their Capacities for Cadmium Removal from Cadmium-Polluted Water
Source: PLoS One. 2016 Apr 14;11(4):e0151479. doi: 10.1371/journal.pone.0151479 (PMC4831789; doi:10.1371/journal.pone.0151479)
Supplement: S1 Table — (DOCX) [file pone.0151479.s003.docx]

**S1 Table. Virulence genes using primers and its parameters**

| ***Primes*** | **Base sequence** | **Product size** | **Annealing temperature (T)** |
| --- | --- | --- | --- |
| ***plcR-F*** | CTATTATTATATGTGAGATGAATTGTATGGTAA | 700 | 55 |
| ***plcR-R*** | AGACGTTTGGATGTTACTCC |  |  |
| ***hblA-F*** | GTGCAGATGTTGATGCCGAT | 320 | 55 |
| ***hblA-R*** | ATGCCACTGCGTGGACATAT |  |  |
| ***HblC-F*** | AATCAAGAGCTGTCACGAAT | 750 | 55 |
| ***HblC-R*** | CACCAATTGACCATGCTAAT |  |  |
| ***hblD-F*** | AATGGTCATCGGAACTCTAT | 430 | 55 |
| ***hblD-R*** | CTCGCTGTTCTGCTGTTAAT |  |  |
| ***nheA-F*** | TACGCTAAGGAGGGGCA | 500 | 55 |
| ***nheA-R*** | GTTTTTATTGCTTCATCGGCT |  |  |
| ***nheB-F*** | CTATCAGCACTTATGGCAG | 770 | 55 |
| ***nheB-R*** | ACTCCTAGCGGTGTTCC |  |  |
| ***nheC-F*** | CGGTAGTGATTGCTGGG | 583 | 55 |
| ***nheC-R*** | CAGCATTCGTACTTGCCAA |  |  |
| ***bceT-F*** | CGTATCGGTCGTTCACTCGG | 661 | 55 |
| ***bceT-R*** | GTTGATTTTCCGTAGCCTGGG |  |  |
| ***Ces-F*** | GGTGACACATTATCATATAAGGTG | 1271 | 58 |
| ***Ces-R*** | GTAAGCGAACCTGTCTGTAACAACA |  |  |
