# Supplementary material for: Cd-Resistant Strains of B. cereus S5 with Endurance Capacity and Their Capacities for Cadmium Removal from Cadmium-Polluted Water
Source: PLoS One. 2016 Apr 14;11(4):e0151479. doi: 10.1371/journal.pone.0151479 (PMC4831789; doi:10.1371/journal.pone.0151479)
Supplement: S2 Table — (DOCX) [file pone.0151479.s004.docx]

**S2 Table. Body weights of mice after direct gavage and drinking of the *B. cereus* S5 strain (unit: g).**

| **Group** | **Sample** | **Body weight of mice (g)** | | | | | | | | |
| --- | --- | --- | --- | --- | --- | --- | --- | --- | --- | --- |
|  |  | 0th | 3^th^ | 6^th^ | 8^th^ | 12^th^ | 16^th^ | 21^th^ | 24^th^ | 27^th^ |
| **Group (CK)** | 1 | 37.4 | 40.1 | 39.8 | 39.8 | 42.1 | 44 | 44.3 | 44.9 | 43.5 |
|  | 2 | 36.8 | 38.4 | 41 | 40.8 | 40.7 | 42.3 | 41.3 | 45.1 | 42.7 |
|  | 3 | 38.1 | 39.8 | 41.2 | 42.2 | 40.8 | 41.1 | 41.4 | 44.6 | 44.7 |
|  | 4 | 39.4 | 40.4 | 41.3 | 40.4 | 40.7 | 42.2 | 45.2 | 43.6 | 42.9 |
|  | 5 | 38.7 | 39.8 | 41 | 42 | 42.4 | 41.9 | 42.8 | 42.7 | 44.7 |
|  | Total | 190.4 | 198.5 | 204.3 | 205.2 | 206.7 | 211.5 | 215 | 220.9 | 218.5 |
|  | Average | 38.08 | 39.7 | 40.86 | 41.04 | 41.34 | 42.3 | 43 | 44.18 | 43.7 |
| **Group (A)** | 1 | 38.3 | 41 | 41.4 | 42.5 | 43.1 | 43.1 | 47.5 | 58.3 | 48.1 |
|  | 2 | 39.8 | 42.3 | 41.3 | 42.4 | 42.2 | 43 | 54.2 | 48.2 | 57.2 |
|  | 3 | 42.5 | 40.4 | 42.8 | 47.6 | 45.3 | 46.5 | 44.2 | 44.5 | 45.4 |
|  | 4 | 39.5 | 44.8 | 45.3 | 44.5 | 49.6 | 52.3 | 47 | 47.1 | 47.3 |
|  | Total | 160.1 | 168.5 | 170.8 | 177 | 180.2 | 184.9 | 192.9 | 198.1 | 198 |
|  | Average | 40.03 | 42.13 | 42.7 | 44.25 | 45.05 | 46.23 | 48.23 | 49.53 | 49.5 |
| **Group (B)** | 1 | 39.6 | 39.2 | 40.8 | 50 | 50.4 | 51.3 | 54.6 | 48.2 | 48.2 |
|  | 2 | 37.9 | 41.3 | 43.3 | 43.4 | 43.8 | 44.6 | 44.6 | 47.3 | 46.8 |
|  | 3 | 39.1 | 42.2 | 48.3 | 41.7 | 41 | 42 | 46 | 47 | 47.1 |
|  | 4 | 43.2 | 46 | 42.4 | 43.5 | 42.7 | 43.6 | 46.8 | 56.2 | 56.5 |
|  | Total | 159.8 | 168.7 | 174.8 | 178.6 | 177.9 | 181.5 | 192 | 198.7 | 198.6 |
|  | Average | 39.95 | 42.18 | 43.7 | 44.65 | 44.48 | 45.38 | 48 | 49.68 | 49.65 |
